# Prevalence of Workplace Bullying and Risk Groups in Chinese Employees in Hong Kong

**DOI:** 10.3390/ijerph18010329

**Published:** 2021-01-05

**Authors:** Catalina Sau Man Ng, Victor C. W. Chan

**Affiliations:** Department of Early Childhood Education, Education University of Hong Kong, Hong Kong, China; chunwa@eduhk.hk

**Keywords:** workplace bullying, prevalence, sociodemographic variables, epidemiology, Chinese employees

## Abstract

Most studies on workplace bullying have been conducted in high-income countries and on Caucasian samples. Little is known about workplace bullying in Asian countries despite its recognition as a serious public health issue in the workplace. We examined the annual and lifetime prevalence of workplace bullying and its risk factors among Chinese employees in Hong Kong. The study was part of a larger project consisting of two waves. Respondents were recruited from a convenience sampling technique and completed a self-reported survey. Respondents reported whether they had been bullied at work for the past 12 months and during their lifetime. A multivariate logistic regression was conducted to explore the sociodemographic risk factors for workplace bullying. There were a total of 2657 respondents (54.6% male), with a mean age of 41.53 years. The annual and lifetime prevalence of workplace bullying were 39.1% and 58.9%, respectively. Multivariate analyses showed that workplace bullying in the past 12 months was associated with a high monthly income, and the combination of a high monthly income and higher educational attainment was associated with bullying at some point in the participants’ career. Suitable policies and interventions to reduce the extent of workplace bullying in Hong Kong are warranted.

## 1. Introduction

Workplace bullying has become a significant public health issue. Over 90% of the studies on workplace bullying come from high-income countries, particularly Europe, [1,2] and have used Caucasian samples. There is a paucity of evidence about workplace bullying in Asian countries [3]. In Hong Kong, the only available data regarding workplace bullying among Chinese employees are limited to a telephone survey conducted in 2013 by an employer service consultancy which found that 53% of the 509 Chinese respondents had been victims of at least one type of workplace bullying [4]. This warrants the need for updated empirical data to provide researchers and policy makers with a complete overview of the prevalence of workplace bullying in Hong Kong.

Research on bullying behavior is based on two major approaches: the self-labelling approach (i.e., whether the respondents perceive themselves as being bullied), and the behavioral experience approach (i.e., based on valid measurements) [5]. Nielsen and colleagues (2010) reported that the prevalence rates of workplace bullying using the self-labelling method with and without a given definition of bullying were 11.3% and 18.1%, respectively, while the result from the behavioral experience approach was 14.8% [6]. The prevalence rates of workplace bullying vary substantially depending on the definition of bullying, how the questions were asked, and cultural or geographical characteristics [7,8,9].

The existing literature providing insights into characteristics of the risk groups that are likely to be victims of workplace bullying is divided and inconclusive. Females were more likely to be bullied than males in some studies [10,11,12] while many studies reported no significant gender differences [13,14,15,16]. Regarding the association between age and workplace bullying, the results were also mixed. Many studies showed no association [17,18], while Hoel and Cooper (2000) reported that younger employees experienced more workplace bullying than older ones [14]. Feijó, Gräf, Pearce, and Fassa (2019) specifically concluded that workers younger than 44 years old were more likely to be bullied [1]. In contrast, two earlier studies [19,20] consistently reported that older workers were at a greater risk of being bullied compared with younger ones. The results on the relationship between workplace bullying and educational qualifications are conflicting. A systematic review found that there was no association between educational level and workplace bullying [1], whereas there was a strong association between bullying and educational level in some studies [21,22,23]. These inconsistent results are not only limited to educational level but also extend to the marital status of employees. Workers who were single regardless of whether they were separated, divorced, or widowed were more likely to be bullied in their workplace [24,25,26] but some studies reported a higher risk of bullying among married workers and those with children [21,27,28]. On the contrary, some studies found no association between workplace bullying and marital status [22,29]. The findings on occupation also yielded different results. Clerks, associate professionals, industrial workers, graphical workers, workers working in hotels and restaurants, and low levels of white- and blue-collar workers for men, and government associate professionals for women reported that they were the highest risk groups [20,30,31]. Based on the results of the socio-demographic risks of workplace bullying, our existing knowledge has been limited to the findings from Western countries and knowledge of the high risk groups in the Asian context are lacking. Furthermore, to enable researchers and policy makers to better design intervention programs in accordance with particular characteristics and the needs of these groups, studies exploring the socio-demographic characteristics of the risk groups of workplace bullying are needed.

The objectives of the present study were: (1) to assess the annual and lifetime prevalence of workplace bullying among Chinese employees in Hong Kong, and (2) to identify the risk groups of workplace bullying, with particular reference to sociodemographic characteristics (gender, age, education, and occupation).

## 2. Methods

The present study was part of a larger longitudinal study which investigated whether victims of workplace bullying affected their children’s health, behaviors, and school adjustment via parenting. The details of the study have been published elsewhere [32]. The data of respondents were collected from 21 elementary schools across the 18 districts covering the three regions of Hong Kong (Hong Kong Island, Kowloon, and the New Territories). The present study reported the cross-sectional data of the parent respondents from Wave 1 regarding whether they have experienced workplace bullying.

### 2.1. Respondents 

The inclusion criteria of the respondents were: (i) Chinese; (ii) aged from 18 (the legal age to work in Hong Kong) to 60 years old; (iii) currently working either full-time or part-time. The exclusion criterion was: individuals who were stay-at-home mothers or fathers or jobless or retired at the time of completing the questionnaires. We used a convenience sampling technique for recruiting respondents from these 21 primary schools.

### 2.2. Procedures

We first sent emails to a randomly selected elementary school in each district based on the list of schools prepared earlier, followed by phone calls to school principals to elaborate on the objectives of the study and invite them to join. Once the schools consented to join the study, written consent from parents was sought prior to data collection. In addition to questionnaires, we included a sheet of instructions to consenting parents on how to complete the questionnaires. Both parents were reminded to complete the questionnaire independently, i.e., without discussing with each other. Each parent was instructed to place their own completed questionnaire in a sealed envelope provided. Each child returned two sealed envelopes to class teachers and we collected all the parent questionnaires from the schools. The anonymity and confidentiality of data as well as the right to withdraw from the study at any time by the respondents without any consequences were included in the instructions. The procedures of data collection followed closely the ethics procedures set by the University’s Human Research Ethics Committee (HREC). 

### 2.3. Measures

Parent respondents completed a self-administered and anonymous questionnaire in Chinese. At the time of data collection, all the instruments used for the present study were available in Chinese.

#### 2.3.1. Questionnaire

##### Socio-Demographic Information

A section of the questionnaire collected information about the respondents’ qualifications/educational level, occupation, current mode of employment (full-time, part-time, others (stay-at-home dad/mum, unemployment, retired), hours per week in the contract, actual hours per week, individual income, and number of days of paid leave. Data for age, gender, marital status, number of children, and district of residence were also collected. 

The part on qualifications/educational level was divided into five categories from primary schooling or below to master’s degree or above. The part on occupations was divided into ten categories: managers and administrators; professionals; associate professionals; clerical support workers; service and sales workers; craft and related workers; plant and machine operators and assemblers; unskilled workers; disciplinary force personnel; and others. The classification of occupations was based on the categorization by the Hong Kong Census and Statistics Department. 

##### Annual and Lifetime Prevalence of Workplace Bullying 

The annual and lifetime prevalence of workplace bullying was assessed by the two single items. The respondents were asked whether they had experienced bullying in their workplace over the past 12 months: “Have you ever been bullied at work over the past 12 months?”. This self-identified bullying was commonly used in the existing literature e.g., [19,33]. The respondents selected one of the following four options: no, never bullied/yes, seldom/yes, sometimes/yes, often. A similar question on the lifetime prevalence of workplace bullying was asked: “Have you ever been bullied at work in your life?”. The same options were given to the respondents. The respondents who indicated ‘no’ were classified as ‘never bullied’, while those who indicated ‘yes’ and rated ‘seldom’, ‘sometimes’, or ‘often’ were categorized as ‘bullied. Prior to the completion of two self-identification items for annual and lifetime prevalence, the respondents completed the Chinese Workplace Bullying Scale (CWBS) [34] so they would have knowledge of workplace bullying and bullying behaviors. 

### 2.4. Statistical Analysis

All statistical analyses were performed using SPSS version 25.0 for Windows (IBM Corporation, Armonk, NY, USA). Descriptive statistics including frequencies and percentages were used to describe the characteristics of the respondents. χ^2^ tests were used to compare categorical variables for the differences between group frequencies. The prevalence of workplace bullying was assessed and presented in terms of frequency and the proportion of those experiencing bullying at work. Prevalence estimates (%) were presented at 95% confidence intervals (CI) calculated from the standard error. We performed a multivariate logistic regression to explore which variables in the model contributed to workplace bullying. The set of risk factors (independent variables) included those variables found significant in χ^2^ tests. Then we used the standard method of entry by entering all independent variables and covariates into the equation at the same time. The adjusted odds ratios (AOR) whose value has been adjusted for the other covariates, including confounders and the corresponding 95% CIs for OR were calculated. Through this paper, data were based on valid responses for each group since not all respondents answered all questions. All reported *p*-values are 2-tailed with statistical significance set at 0.05. 

## 3. Results

We sent out 3600 questionnaires and a total of 2657 Chinese respondents completed the questionnaires, at a response rate of 73.8%. 

### 3.1. Socio-Demographic Characteristics of the Respondents

There were 2657 Chinese respondents (54.6% male, 45.4% female; M_age_ = 41.53 years, SD = 5.69, age range = 19 to 60 years). Of the 2649 respondents who provided valid responses on education, over half of the respondents received secondary education (52.2%), followed by post-secondary education (17.9%), bachelor’s degree (17.4%), master’s degree or above (9.6%) and primary education or below (3.0%). Of the 2634 respondents with valid responses on marital status, 93.3% were married. The sociodemographic characteristics of the respondents are summarized in Table 1.

### 3.2. Annual and Lifetime Prevalence of Workplace Bullying

Respondents who were unemployed, retired, stay-at-home fathers or housewives were excluded in the analyses. Only those who indicated their gender, were aged between 18 and 60 years old, were currently working either full-time or part-time, and answered the questions regarding having experienced workplace bullying in the past 12 months and during their lifetime were included from the analysis. A total of 1018 out of 2607 valid responses indicated that the respondents sometimes or often experienced workplace bullying in the past 12 months; 1550 out of 2631 valid responses indicated that the respondents have experienced workplace bullying in their lifetime. The annual and lifetime prevalence rates of workplace bullying were 39.1% (95% CI: 37.2% to 41.0%) and 58.9% (95% CI: 57.0% to 60.8%), respectively. 

### 3.3. Characteristics of the Risk Groups of Workplace Bullying

Table 2 reports the demographic characteristics of respondents who reported being never bullied and bullied in the previous 12 months and in their lifetime. There was no statistical difference between genders for annual prevalence (39.0% male vs. 39.1% female, *p* = 0.986) and lifetime prevalence (57.6% male vs. 60.5% female, *p* = 0.144) of workplace bullying. 

For respondents who reported having experienced workplace bullying in the past 12 months, there was no statistically significant difference between age groups (aged 18–30: 27.5% vs. aged 31–40: 40.1% vs. aged 41–50: 39.0% vs. aged 51–60: 36.4%, *p* = 0.273). However, for respondents who reported having experienced workplace bullying during their lifetime, higher prevalence was found in those aged 31 or above as compared to those aged 30 or below (aged 18–30: 35.8% vs. aged 31–40: 59.7% vs. aged 41–50: 59.5% vs. aged 51–60: 57.1%, *p* = 0.006). 

For respondents who reported having experienced workplace bullying in the past 12 months, the prevalence rate increased with the level of education (primary education or below: 33.3% vs. secondary education: 35.8% vs. college education: 40.0% vs. bachelor’s degree: 46.0% vs. master’s degree or above: 44.8%, *p* = 0.001). A similar pattern was found in those who reported having experienced workplace bullying during their lifetime (primary education or below: 39.7% vs. secondary education: 54.2% vs. college education: 60.1% vs. bachelor’s degree: 68.9% vs. master’s degree or above: 70.6%, *p* < 0.001).

For respondents who reported having experienced workplace bullying in the past 12 months, there was no statistical difference between occupation groups (*p* = 0.054). More than 50% of responses for each of the occupation groups indicated that the respondents had never bullied in the past 12 months. For respondents who reported having experienced workplace bullying during their lifetime, over 50% of responses for each of the occupation groups indicated that respondents had experienced workplace bullying (*p* < 0.001). High prevalence rates (more than 60%) were found in the following occupation groups—professionals (67.4%), followed by disciplinary force personnel (65.1%), associate professionals (64.7%), clerical support workers (63.9%), and managers and administrators (61.8%). 

### 3.4. Risk Groups Associated with Workplace Bullying

In the model of multivariate logistic regression (Table 3), increasing monthly individual income (HK $10,000–19,999, Adjusted odds ratios (AOR): 1.17, 95% CI: 0.67–1.58, *p* = 0.309; HK $20,000–29,999, AOR: 1.38, 95% CI: 1.01–1.89, *p* = 0.043; HK $30,000–39,999, AOR: 1.70, 95% CI: 1.22–2.37, *p* = 0.002; ≥HK $40,000, AOR: 1.55, 95% CI: 1.11–2.18, *p* = 0.011) remained a significant independent risk factor for experiencing workplace bullying in the past 12 months. 

For having experienced workplace bullying during their lifetime (Table 4), increasing level of education attainment (secondary education, AOR: 1.36, 95% CI: 0.82–2.28, *p* = 0.237; college, AOR: 1.69, 95% CI: 0.97–2.95, *p* = 0.062; bachelor’s degree, AOR: 2.14, 95% CI: 1.20–3.82, *p* = 0.010; master’s degree or above, AOR: 2.46, 95% CI: 1.31–4.61, *p* = 0.005) and increasing monthly individual income (HK $10,000–19,999, AOR: 1.33, 95% CI: 0.99–1.79, *p* = 0.055; HK $20,000–29,999, AOR: 1.71, 95% CI: 1.24–2.36, *p* = 0.001; HK $30,000–39,999, AOR: 2.20, 95% CI: 1.52–3.19, *p* < 0.001; ≥HK $40,000, AOR: 1.73, 95% CI: 1.20–2.51, *p* = 0.004) were shown as significant independent risk factors.

## 4. Discussion

The present study aimed to assess the annual and lifetime prevalence and the risk groups of workplace bullying in 2657 Chinese employees in Hong Kong. The total prevalence of workplace bullying was 39.1% and 58.9% for the last 12 months and at some point in their working life, respectively. In Ciby and Raya’s (2015) review, they reported that the highest prevalence of workplace bullying was found in Asia (over 52% in Turkey and Pakistan) [35]. Although it is difficult to compare the prevalence rates due to the use of different measurement methods, tools, and operational criteria [36], our results are concerning as the rates are more than double the global prevalence rate of 15% [37,38], suggesting that the issue must be addressed urgently. 

Some possible reasons for this high prevalence are first in the current study, the respondents were asked about whether they have been bullied after the completion of the modified CWBS [34], which provided them with some prior knowledge of bullying behaviors. It is probable that the respondents had some awareness of workplace bullying, so they reported their experience as such. Second, the present study used a convenience sampling technique which many workplace bullying studies have adopted [39]. However, Nielsen [40] highlighted that representative and convenience samples provide significantly different estimates of the prevalence of bullying, with convenience samples resulting in more reports of frequent and intense exposure to bullying. Third, the present study examined the prevalence of workplace bullying over the past 12 months, which is considered one of the most appropriate time frames to use in workplace bullying research [40]. It is worth noting that a study using a longer reporting period will result in a higher prevalence rate than a study using a shorter reporting period [41]. Fourth, as argued by Nielsen, Tangen, Idsoe, Matthiesen, and Magerøy [42], countries with a larger power distance may report a comparatively higher prevalence of workplace bullying. Since power hierarchy is highly emphasized in Hong Kong workplaces and Hong Kong is a place with strong Confucian values which places more emphasis on maintaining social harmony and respecting elders, people are more obedient to and tend to be more accepting of people who have power over others, so it is likely that a higher percentage of workplace bullying may have been reported.

The results from multivariate logistic regression analyses found that a higher monthly personal income was a common significant risk factor for those experiencing workplace bullying in the past 12 months and during their lifetime. Furthermore, having higher education qualifications was an additional risk factor for workplace bullying during the lifetime. Our results from χ^2^ tests supported that professional groups had the highest prevalence rate of bullying at work, which matched the results of regression analyses—employees of professional groups often have higher level of education and better income. However, our findings were inconsistent with most studies which reported that workplace bullying aggregates in socially disadvantaged groups [18,30]. The fact that having postsecondary education or below is a protective factor against negative workplace bullying behaviors [43] is relevant to our study. Specifically, our results showed that having a bachelor’s degree was a risk factor for workplace bullying. One possible explanation is that Chinese employees with a higher education level are more likely to be perceived as rivals or threats by their supervisors or co-workers because of fear or jealousy in a competitive work environment like Hong Kong. For supervisors or co-workers, feelings of insecurity or the fear of being replaced may be aroused if the subordinates or co-workers are high-performing employees. This sense of insecurity might drive supervisors or co-workers to be manipulative such as concealing useful information or overloading the targets with tasks to affect their work performance. Another plausible reason is that those with higher education might behave more aggressively or may adopt certain types of behaviors which lead them to be the targets of bullying. Larson (2013) provided an example stating that the behaviors of younger nurses with a bachelor’s degree sometimes behaved rudely and condescendingly to veteran nurses with diploma who took a longer time to adapt to new technology [44]. 

Contrary to our expectation, our results from regression analyses showed that Chinese employees with higher monthly income were a risk factor for workplace bullying for the past 12 months and during the lifetime. Our results were inconsistent with Bashir, Hanif, and Nadeem’s study [45], which found that income level was not associated with workplace bullying. Our findings were also different from the majority of studies—workers with low and minimum-wage were more likely to be bullied [1,46,47] because their employment is often at the will of the employers. Our finding was consistent with a Malaysian study [48] and Chan and colleagues argued that income levels may not be a clear indicator for risk of workplace bullying even where economic disparities are evident. In the context of Hong Kong, it is likely that those with higher monthly income occupy a better and hierarchically higher position in their workplace. To get a higher position involves a lot of power struggle and office politics, which ranges from bullying to the ultimate elimination of rivals. Another possible explanation is that some organizations would prefer to dismiss those employees with higher salary and higher positions to employ other employees willing to accept a lower salary. Bullying is a means to push those employees away. 

### Limitations and Future Directions 

The present study has provided empirical results from an Asian city which is an area currently under-researched in the field and enjoyed a high participation rate from the respondents. However, several limitations need to be considered in interpreting the results of the present study. First, the present study has used convenience sampling which limits the generalizability of the results. Second, since the definition of workplace bullying was not included in the survey, the annual and lifetime prevalence rates of the present study can be considered tentative as they may be overinflated, resulting from the fact that the respondents reported more incidences than actually happened due to some judgment bias [6]. Third, all variables are based on self-report, so there may have been some socially desirable responses which could confound the research results. However, given the high annual and lifetime prevalence rates, it is less likely that the respondents provided socially desirable responses. Fourth, the respondents were all employees with a Chinese cultural background working in Hong Kong and a sample exclusively comprised of adults with school-age children, so the results are not generalizable to other populations. The findings of the present study need to be replicated in a sample of the general population cross-culturally and in different Chinese communities such as in Mainland China and Taiwan.

## 5. Conclusions

Despite the above limitations, the present findings extend the sparse literature on the prevalence of workplace bullying among Chinese employees in Hong Kong. The results of the present study allow us to identify and understand better the risk groups of workplace bullying in a Chinese context which is currently under-researched. Tailor-made policies and suitable interventions by the government and corporations to reduce bullying at work are warranted to eliminate this phenomenon.

## Figures and Tables

**Table 1:** Sociodemographic characteristics of the respondents (*n* = 2657).

Demographic Variables	*n* (%)
Gender (*n* = 2657)	
Male	1450 (54.6)
Female	1207 (45.4)
Educational attainment (*n* = 2649)	
Primary education or below	79 (3.0)
Secondary education	1383 (52.2)
College	473 (17.9)
Bachelor’s degree	461 (17.4)
Master’s degree or above	253 (9.6)
Age (years) (*n* = 2657)	
18–30	55 (2.1)
31–40	1106 (41.6)
41–50	1330 (50.1)
51–60	166 (6.2)
Marital status (*n* = 2634)	
Married/cohabitation	2483 (94.3)
Separated/divorced/widowed/others	151 (5.7)
Number of children (*n* = 2653)	
1	875 (33.0)
2	1488 (56.1)
3 or more	290 (10.9)
Occupational group (*n* = 2464)	
Craft and related workers	176 (7.1)
Clerical support workers	378 (15.3)
Associate professionals	150 (6.1)
Service and sales workers	374 (15.2)
Managers and administrators	495 (20.1)
Unskilled workers	116 (4.7)
Professionals	358 (14.5)
Disciplinary force personnel	106 (4.3)
Plant and machine operators and assemblers	114 (4.6)
Others	197 (8.0)
Monthly individual income (HK$) (*n* = 2451)	
≤9999	282 (11.5)
10,000–19,999	715 (29.2)
20,000–29,999	528 (21.5)
30,000–39,999	386 (15.8)
≥40,000	540 (22.0)

Note: Due to rounding, the total percentage may not add up to 100%.

**Table 2:** Demographic characteristics of respondents and exposure to workplace bullying.

	Reported Workplace Bullying in the Past 12 Months				Reported Workplace Bullying during Lifetime			
Characteristics	Never Bullied *n* (row%)	Ever Bullied *n* (row%)	χ^2^ *p*-Value	Phi/ Cramer’s V	Never Bullied *n* (row%)	Ever Bullied *n* (row%)	χ^2^ *p*-Value	Phi/ Cramer’s V
All	1589 (61.0)	1018 (39.0)			1081 (41.1)	1550 (58.9)		
Gender			0.986	<.001			0.144	0.03
Male	870 (61.0)	557 (39.0)			610 (42.4)	830 (57.6)		
Female	719 (60.9)	461 (39.1)			471 (39.5)	720 (60.5)		
Educational attainment			0.001	0.09			<0.001	0.15
Primary education or below	52 (66.7)	26 (33.3)			47 (60.3)	31 (39.7)		
Secondary education	868 (64.2)	484 (35.8)			627 (45.8)	743 (54.2)		
College	277 (60.0)	185 (40.0)			186 (39.9)	280 (60.1)		
Bachelor’s degree	248 (54.0)	211 (46.0)			142 (31.1)	315 (68.9)		
Master’s degree or above	137 (55.2)	111 (44.8)			74 (29.4)	178 (70.6)		
Age (years)			0.273	0.04			0.006	0.07
18–30	37 (72.5)	14 (27.5)			34 (64.2)	19 (35.8)		
31–40	648 (59.9)	434 (40.1)			441 (40.3)	652 (59.7)		
41–50	799 (61.0)	510 (39.0)			536 (40.5)	786 (59.5)		
51–60	105 (63.6)	60 (36.4)			70 (42.9)	93 (57.1)		
Marital status			0.801	0.01			0.792	0.01
Married/cohabitation	1485 (60.9)	953 (39.1)			1006 (40.9)	1453 (59.1)		
Separated/divorced/widowed/others	88 (59.9)	59 (40.1)			63 (42.0)	87 (58.0)		
Number of children			0.499	0.02			0.126	0.04
1	514 (59.6)	349 (40.4)			341 (39.2)	528 (60.8)		
2	898 (61.4)	565 (38.6)			606 (41.3)	863 (58.7)		
3 or more	175 (63.2)	102 (36.8)			133 (46.0)	156 (54.0)		
Occupational group			0.054	0.08			<0.001	0.12
Craft and related workers	103 (60.9)	66 (39.1)			81 (47.1)	91 (52.9)		
Clerical support workers	239 (63.7)	136 (36.3)			135 (36.1)	239 (63.9)		
Associate professionals	78 (53.8)	67 (46.2)			53 (35.3)	97 (64.7)		
Service and sales workers	231 (62.9)	136 (37.1)			177 (48.0)	192 (52.0)		
Managers and administrators	299 (61.3)	189 (38.7)			187 (38.2)	303 (61.8)		
Unskilled workers	70 (63.1)	41 (36.9)			51 (45.1)	62 (54.9)		
Professionals	194 (54.3)	163 (45.7)			116 (32.6)	240 (67.4)		
Disciplinary force personnel	55 (51.9)	51 (48.1)			37 (34.9)	69 (65.1)		
Plant and machine operators or assemblers	73 (64.6)	40 (35.4)			55 (48.2)	59 (51.8)		
Others	123 (64.4)	68 (35.6)			92 (46.9)	104 (53.1)		
Monthly individual income (HK$)			<0.001	0.10			<0.001	0.13
≤9999	185 (68.3)	86 (31.7)			145 (52.5)	131 (47.5)		
10,000–19,999	457 (65.0)	246 (35.0)			320 (45.5)	384 (54.5)		
20,000–29,999	317 (60.2)	210 (39.8)			202 (38.4)	324 (61.6)		
30,000–39,999	206 (54.4)	173 (45.6)			125 (32.6)	259 (67.4)		
≥40,000	295 (55.3)	238 (44.7)			184 (34.3)	352 (65.7)		

**Table 3:** Adjusted odds ratios (AOR) and 95% confidence intervals (95% CI) of workplace bullying in the past 12 months by risk factors.

	Reported Workplace Bullying in the Past 12 Months	
Characteristics	AOR (95% CI)	*p*-Value
Educational attainment		0.235
Primary education or below	1 (reference)	
Secondary education	0.98 (0.59–1.63)	0.935
College	1.16 (0.68–1.99)	0.583
Bachelor’s degree	1.28 (0.74–2.21)	0.376
Master’s degree or above	1.15 (0.64–2.07)	0.639
Monthly individual income (HK$)		0.010
≤9999	1 (reference)	
10,000–19,999	1.17 (0.67–1.58)	0.309
20,000–29,999	1.38 (1.01–1.89)	0.043
30,000–39,999	1.70 (1.22–2.37)	0.002
≥40,000	1.55 (1.11–2.18)	0.011

**Table 4:** Adjusted odds ratios (AOR) and 95% confidence intervals (95% CI) of workplace bullying during lifetime by risk factors.

	Reported Workplace Bullying during Lifetime	
Characteristics	AOR (95% CI)	*p*-Value
Educational attainment		0.004
Primary education or below	1 (reference)	
Secondary education	1.36 (0.82–2.28)	0.237
College	1.69 (0.97–2.95)	0.062
Bachelor’s degree	2.14 (1.20–3.82)	0.010
Master’s degree or above	2.46 (1.31–4.61)	0.005
Age (years)		0.379
18–30	1 (reference)	
31–40	1.62 (0.86–3.05)	0.133
41–50	1.47 (0.78–2.77)	0.235
51–60	1.50 (0.74–3.06)	0.262
Occupational group		0.219
Craft and related workers	1.06 (0.69–1.63)	0.792
Clerical support workers	1.56 (1.08–2.24)	0.017
Associate professionals	1.28 (0.81–2.02)	0.301
Service and sales workers	1.22 (0.85–1.76)	0.280
Managers and administrators	1.03 (0.72–1.48)	0.876
Unskilled workers	1.55 (0.95–2.55)	0.080
Professionals	1.20 (0.80–1.79)	0.387
Disciplinary force personnel	1.19 (0.71–2.02)	0.509
Plant and machine operators or assemblers	1.02 (0.63–1.65)	0.924
Others	1 (reference)	
Monthly individual income (HK$)		0.001
≤9999	1 (reference)	
10,000–19,999	1.33 (0.99–1.79)	0.055
20,000–29,999	1.71 (1.24–2.36)	0.001
30,000–39,999	2.20 (1.52–3.19)	<0.001
≥40,000	1.73 (1.20–2.51)	0.004

## Data Availability

Data available on request due to ethical considerations. The data presented in this study are available on request from the corresponding author.
